# Gender Disparities in First Authorship in Publications Related to Attention Deficit Hyperkinetic Disorder (ADHD) and Artificial Intelligence (AI)

**DOI:** 10.7759/cureus.49714

**Published:** 2023-11-30

**Authors:** Jeby Abraham, Kashyap Panchal, Leena Varshney, Kiran Lakshmi Narayan, Saman Rahman

**Affiliations:** 1 General Medicine, Yenepoya Medical College, Mangalore, IND; 2 Psychiatry and Behavioral Sciences, American University of Barbados, St. Michael, BRB; 3 Preventive Medicine, Windsor University School of Medicine, Troy, USA; 4 Pediatrics, Aasare Hospital, Bengaluru, IND; 5 Internal Medicine, Jawaharlal Nehru Medical College, Belagavi, IND

**Keywords:** gender parity, inclusivity, gender equality, gender trends, publications, artificial intelligence, adhd, first authors, gender disparities

## Abstract

The medical profession has experienced a significant increase in the number of women practitioners in recent decades, leading to a reduction in the gender gap. According to the United States Medical Association, approximately 25% of physicians in the United States are now women. Although this progress is evident in the clinical setting, women's representation in academic medicine remains disproportionately low. The underrepresentation of women in academia has various consequences, including limited access to academic resources and hindered career growth. Previous studies have attempted to analyze these disparities, but results have been inconsistent, and the issue's complexity has not been fully understood. This study aims to examine the disparity in the gender of first authors in academic publications related to " Artificial intelligence (AI) and Attention Deficit Hyperkinetic Disorder (ADHD)" between 2010 and 2023.

Analysis was conducted on June 21st, 2023, using the database PubMed. The search term "AI" AND "ADHD" was used to derive all articles over a period of 13 years, from January 1st, 2010, to December 31st, 2022, excluding the year 2023 due to limited available publications. The relevant articles were downloaded in Microsoft Excel sheets. The gender of the first authors was determined using the NamSor app V.2, an application programming interface (API) with a large dataset of names and countries of origin.

A total of 204 articles were considered for this study. There were 78 female first authors and 126 male first authors. The highest number of publications with a male first author occurred in 2022, with 32 publications. The Netherlands, Singapore, Turkey, and China have the highest gender ratios, indicating a more favourable representation of both genders. The p-value of 0.2664 suggests that there is no significant association between gender and country.

The findings revealed a gender disparity, with a higher number of male first authors. By addressing and rectifying these disparities, we can enhance the overall quality, diversity, and inclusivity of research in the field of ADHD and Artificial Intelligence.

## Introduction and background

The evolving landscape of the medical profession has witnessed a growing number of women practitioners over the past decades, causing a shift towards closing the gender gap. According to the United States Medical Association, approximately 25% of physicians in the United States are now women [1]. While this demonstrates the strides women have made in the clinical setting, their representation in academic medicine remains disproportionately low [2].

The underrepresentation of women in academia has multifaceted repercussions, from impacting the allocation of crucial academic resources to restraining their individual career growth [3]. Furthermore, it can impede the advancement of medical knowledge. Academic publications, productivity indicators, and influencers of academic promotions reflect this imbalance. Therefore, examining the gender distribution of first authors in research, particularly beyond the US borders, can offer invaluable insights into gender disparities within the medical field. Although previous studies have sought to analyse these disparities, results have been inconsistent, and the complex nature of the issue is yet to be fully understood [4]. Moreover, limited research has been conducted to assess the gender distribution of first authors in academic articles beyond the United States, underscoring the need for a more comprehensive, global investigation [5-7].

This study seeks to bridge this gap by analyzing gender trends in the first authorship of academic articles focusing on Attention Deficit Hyperactivity Disorder (ADHD) and Artificial Intelligence (AI). This examination spans PubMed-indexed publications from 2010 to 2022 and explores these trends based on country and year.

## Review

This is a bibliometric analysis conducted on June 21st, 2023. The analysis was carried out using the search engine PubMed. Articles pertaining to “ADHD and AI” were analysed to ascertain the gender of the first author, to find out gender trends, and to predict the upcoming trend. PubMed database was searched using these terms: “ADHD" AND "AI.” The articles from the last 13 years, 1st January 2010 to 31st December 2022, were included in this study. Publications from the year 2023 were excluded since only a few publications of five months were available. However, articles accepted in the year 2022 and published and appearing on PubMed in 2023, were included in the study.

All the relevant articles were downloaded as a CSV file. There was a total of 204 entries which were equally divided among five authors. Each author scrutinised the articles for their relevance to the aim of our study. All the data was entered into a Google spreadsheet. Each entry was completed to include PubMed Identifier (PMID), Title, Citation, Journal, Year of publication, digital object identifier (DOI), and PubMed Central identifier (PMCID)/National Institutes of Health Manuscript Submission (NIHMS) ID (if available). Then subsequently, the full name (first and last name), country, and university/institute of the first author were completed via PubMed.

The full name and country (where publication was done) of the first author were used to find out their gender. Gender analysis was limited to first authors only because they contribute more to the paper, and are generally the ones to come up with the idea. NamSor app V.2 (www.Namsor.app), an application programming interface (API), was used for this study [8]. NamSor was selected to find out the gender of the first author because of its large dataset (7.5 billion names); also it uses both name and country of origin for predicting gender. NamSor has a proven accuracy of 97%, is partially free to use, and is easy to learn for beginners. Quality inferential is also provided by NamSor which was not used for this study. To settle any discrepancies a simple internet search was carried out to confirm the gender. Statistical analysis was done using R software version 4.3.2 (R Foundation for Statistical Computing, Vienna, Austria), the AutoRegressive Integrated Moving Average (ARIMA) model, and graphs were prepared using DataWrapper (www.datawrapper.de).

**Figure 1:**
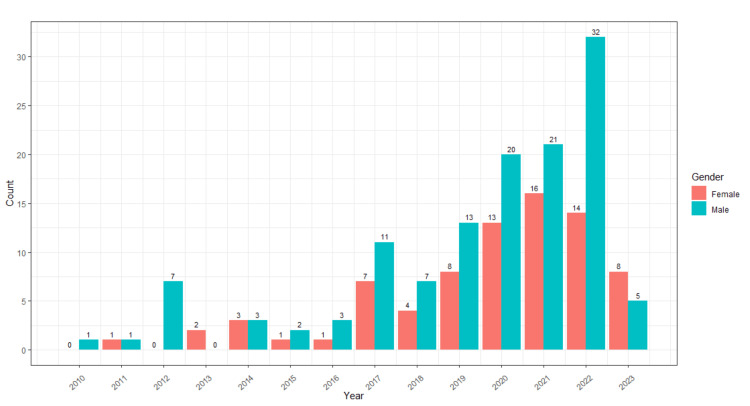
The total number of male and female first authors based on the years ranging from 2010 to 2023.

In this study, a total of 204 doctors participated. 204 doctors' full first names were extracted. So, out of 204, there were 78 female first authors and 126 male first authors. Figure 1 shows the total number of male and female first authors based on the years ranging from 2010 to 2023. The maximum number of publications as a male first author was 32 in the year 2022.

**Figure 2:**
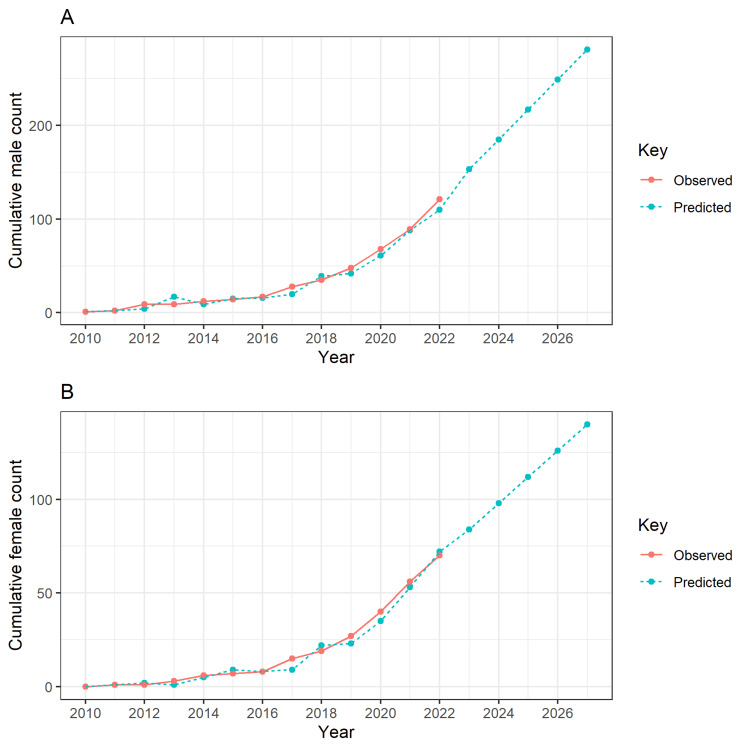
The observed publications on trends among cumulative male count and female count first authors from 2005 to 2022 and predictions of trends for the next five years 2a: Cumulative male first author trend forecasting; 2b: Cumulative female first author trend forecasting.

Figure 2 shows observed publications on trends among cumulative male count and female count first authors from 2005 to 2022 and predictions of trends for the next five years. The modelling data was taken from 2010 to 2022. It is expected that in the year 2026, there will be 250 publications with males as the first authors and 150 publications with females as the first authors. The statistical model used here was the ARIMA model.

**Figure 3:**
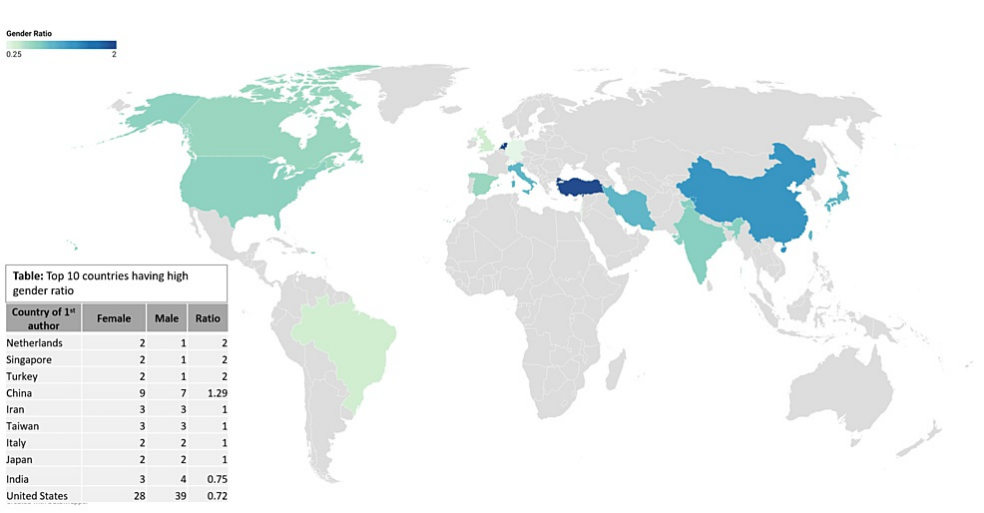
The gender trend in publications based on countries The highest gender ratio is most favourable in the Netherlands, Singapore, and Turkey, followed by China.

Figure 3 shows the gender trend in publications based on countries The highest gender ratio is most favourable in Netherlands, Singapore Turkey, followed by China.

Table 1 shows the top 12 journals with a favourable gender ratio.

**Table 1:** Top journals having a favourable gender ratio

Journal.Book	Female	Male	Ratio	Total
Addict Behav	1	1	1	2
Artif Intell Med	1	1	1	2
Autism Res	1	1	1	2
Child Psychiatry Hum Dev	1	1	1	2
Clin Neurophysiol	1	1	1	2
Comput Biol Med	1	1	1	2
Front Comput Neurosci	1	1	1	2
Front Neurosci	1	1	1	2
J Atten Disord	3	3	1	6
Jmir Res Protoc	1	1	1	2
Neuropsychopharmacology	1	1	1	2
Neuroscience	1	1	1	2

Table 2 shows the top countries showing a high gender ratio with more than five authors and publications in total. In this data, the United States has 28 female publications as first authors, followed by China with nine female publications with a p-value of 0.2664, which shows that there is no significant association between gender and country.

**Table 2:** Top Countries having a high gender ratio (greater than 5 publications)

Country.of.1st.author	Female	Male	Ratio
China	9	7	1.29
Iran	3	3	1
Taiwan	3	3	1
India	3	4	0.75
United States	28	39	0.72
Canada	4	6	0.67
Spain	2	3	0.67
United Kingdom	3	7	0.43
Brazil	2	5	0.4

Table 3 shows the top 10 journals with a high gender ratio. In the data that we collected, Neuroimage: Clinical Journal has the highest gender ratio of 1.5, followed by the rest of the data collection.

**Table 3:** Top journal having a high gender ratio Addict Behav: Addictive Behaviors; Artif Intell Med: Artificial Intelligence in Medicine; Autism Res: Autism Research; Child Psychiatry Hum Dev: Child Psychiatry & Human Development; Clin Neurophysiol: Clinical Neurophysiology; Comput Biol Med: Computers in Biology and Medicine; Front Comput Neurosci: Frontiers in Computational Neuroscience; Front Neurosci: Frontiers in Neuroscience; J Atten Disord: Journal of Attention Disorders; Jmir Res Protoc: JMIR Research Protocols; Neuroimage Clin: NeuroImage: Clinical

Journal.Book	Female	Male	Ratio
Neuroimage Clin	3	2	1.5
J Atten Disord	3	3	1
Addict Behav	1	1	1
Artif Intell Med	1	1	1
Autism Res	1	1	1
Child Psychiatry Hum Dev	1	1	1
Clin Neurophysiol	1	1	1
Comput Biol Med	1	1	1
Front Comput Neurosci	1	1	1
Front Neurosci	1	1	1

Analysing the gender equality trends in this study amongst the first author reveals a concerning gender disparity. Out of the 204 authors included in this study, there is a significant underrepresentation of female first authors, only 78 out of 204 (38.2%). This shows that there is gender inequity within this field of study, with the male-to-female author ratio being 1:1.61.

Upon examining the temporal trends, in 2022, the number of male first-author publications reached its peak, whereas there is no such specific trend for female first authors. Furthermore, using the future predictions from the ARIMA model, this gender disparity is likely to be persistent, as male first-author publications are predicted to be even higher in 2026.

On a positive note, Turkey, the Netherlands, China, and Singapore had favourable gender ratios, suggesting a higher female first-author representation from these regions, as well as, multiple journals displaying favourable gender ratios. Even though there is no statistically significant relationship between gender and the country of publication, suggesting that other more complex social and systemic factors rather than geographic factors are what are driving gender disparity, more needs to be done to ensure that female researchers across the globe have equal opportunities.

Like this study, another study done by Nasrullah et al. (2023), analysing the gender differences in pulmonary and critical care authorships, demonstrated that female first-authors comprised 29.1% of the total authorship [9]. Similarly, Cannon et al. (2020) concluded that out of 448 first and senior authorships, only 10% of them were female in articles published in the Journal of Paediatric Urology [10].

In contrast to the predictions using the ARIMA model, where the female-to-male ratio in 2026 is still low (0.6), other studies done by Madden et al. (2021), examining gender bias in medical education journals, found an increase in female first-authors, from 6.6% in 1970 to 53.7% in 2019 [11]. Another gender gap study by Feramisco et al. (2009), analysing dermatology journals over three decades, found an increase from 12% to 48% in US-affiliated female first-authors from 1976 to 2006 [12].

Likewise, a study by Gervasio et al. (2022) shows an increase in female first-authorship in articles published in Ophthalmic Plastic and Reconstructive Surgery (OPRS) from 3.9% in 1985 to 44.6% in 2020; despite this significant increase, the total percentage of female first-authors was 29.7% [13]. This is highly promising, suggesting an increase in female authorship over the coming years despite current trends.

In contrast to this study, where no significant gender disparity was found in the analysed journals, a systematic review examining the authorship trends in Neuro-oncology in academic journals since 1944 by Behmer Hansen et al. (2022) found that the percentage of female first and last authors in medically oriented journals demonstrated a more noticeable increase rate (0.72% vs. 0.36% per year) in comparison to surgically oriented journals [14].

Contrary to this study, where there was no significant association between gender and countries, a study inspecting Gender equity in Global oncology publications by Hornstein et al. (2022) concluded that out of all the regions, South Asia and sub-Saharan Africa had the lowest percentage of female authors, and especially in low-income countries, female authors made up only 21.6% of the first authors [15].

In a very interesting review by Merriman et al. (2021), studying the differences in gender and geography in leading medical and global health journals, they found that out of the 14 journals examined, only five encouraged the analysis of gender in their authors; however, this did not result in a high gender reporting beyond the gender of study participants [5]. Additionally, women from low- and middle-income countries faced disadvantages in terms of the impact factor of the journals they published in, suggesting inequities based on country income levels [5].

This study has several limitations that should be taken into consideration. This study mainly relies on data obtained from PubMed articles and thus may not include all the studies in the field of “ADHD and Artificial Intelligence,” This could introduce a bias if relevant studies are not included. Only the first authors of the publications were focused upon in this study, which may not provide us with a comprehensive report of gender representation amongst the authorship hierarchy. The limited sample size of 204 authors may be considered a relatively small one, as a larger sample size would provide more accurate and representative results. The 100% accuracy of the Namsor App used cannot be determined. Hence, there is an increased chance of misclassification, potentially impacting the accuracy of this study’s results. Keeping these limitations in mind, further research with larger and more diverse datasets, using more data sources, and including data authorship beyond just the first authors should be conducted to provide a more comprehensive understanding of gender equality trends in publications in “ADHD and Artificial Intelligence.”

## Conclusions

This study indicates the presence of a higher number of males as first authors compared to females in publications related to AI and ADHD. Creating an inclusive environment that not only supports but also encourages female researchers by providing them with equal opportunities will increase the knowledge added to this arena. By addressing and rectifying these disparities, we can enhance the overall quality, diversity, and inclusivity of research in this field.
